# Single‐Nucleus RNA‐Seq Reveals Neuroprotective Effects of Acupuncture in Chronic Migraine Through Modulation of Glial Subtypes

**DOI:** 10.1002/cns.70981

**Published:** 2026-06-16

**Authors:** Shiqi Sun, Shuangyuan Hu, Mingsheng Sun, Zili Tang, Yuyan Wang, Longyao Xu, Puhua Cao, Lu Liu, Rong Wang, Jing Yuan, Ying Chen, Ling Zhao

**Affiliations:** ^1^ Acupuncture and Tuina School Chengdu University of Traditional Chinese Medicine Chengdu Sichuan China; ^2^ Acupoint Effect Key Laboratory of Sichuan Province Chengdu University of Traditional Chinese Medicine Chengdu Sichuan China

**Keywords:** acupuncture, astrocytes, chronic migraine, microglia, single‐nucleus transcriptomics

## Abstract

**Background:**

Chronic migraine (CM) is a debilitating neurological disorder with limited treatment options. Although acupuncture has demonstrated clinical efficacy in relieving CM symptoms, its cellular and molecular mechanisms remain poorly understood.

**Objective:**

This study aimed to delineate the cell‐type‐specific transcriptional landscape and intercellular communication network reshaped by acupuncture.

**Methods:**

A rat model of CM was induced by repeated administration of nitroglycerin. Acupuncture was applied at GB8 and GB34 points. Single‐nucleus RNA sequencing (snRNA‐seq) was performed on the TNC region to profile transcriptomic changes across different cell types. Differential expression analysis, functional enrichment, and pseudotime trajectory inference were performed, along with intercellular communication analysis using CellChat.

**Results:**

Acupuncture alleviated pain‐related behaviors and restored aberrant cell compositions in the TNC, especially among neurons, astrocytes, and microglia. It reversed the CM‐induced upregulation of inflammatory and oxidative stress‐related genes (e.g., *Nfkbia*, *S100a8*, *S100a9*, *Penk*). The imbalance between neuronal excitability and metabolism was rectified through the modulation of glutamatergic transmission and oxidative phosphorylation pathways. Microglial polarization shifted from pro‐inflammatory (M1/M5) to reparative ones (M2/M4), while astrocytic subtypes rebalanced toward anti‐inflammatory and metabolic repair states. CellChat analysis showed that acupuncture also remodeled neuron–glia communication disrupted by CM.

**Conclusions:**

This study sheds light on the cellular and molecular mechanisms by which acupuncture alleviates CM, providing novel insights into its effects on oxidative stress, inflammation, and neuronal function in the TNC. These findings have important implications for both basic science and clinical practice, supporting the potential of acupuncture as a non‐invasive, adjunctive therapy for migraine treatment.

## Graphical abstract

Acupuncture Alleviates Chronic Migraine by Reprogramming Glial Cells and Restoring Neuronal Homeostasis in the Trigeminal Nucleus Caudalis. It suppresses inflammatory and oxidative stress responses, rebalances neuronal excitability and metabolism, drives microglia and astrocytes toward reparative states, and reconstructs impaired neuron–glia communication.

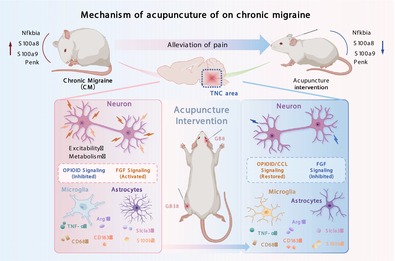

**Abbreviations**
CM: chronic migraine
DEGs: differentially expressed genes
GO: Gene Ontology
GSEA: Gene set enrichment analysis
GSVA: gene set variation analysis
Ipc: intermediate progenitor population
KEGG: Kyoto Encyclopedia of Genes and Genomes
MCC: maximum clique centrality
NTG: nitroglycerin
Oligo: oligodendrocytes
Opc: oligodendrocyte precursor cells
PPI: protein–protein interaction
SD: Sprague–Dawley
snRNA‐seq: single‐nucleus RNA sequencing
TLRs: Toll‐like receptors
TNC: trigeminal nucleus caudalis
TNF‐α: tumor necrosis factor‐alpha
t‐SNE: t‐distributed stochastic neighbor embedding
UMIs: unique molecular identifiers

## 1 Introduction

Chronic migraine (CM) is a highly disabling and heterogeneous neurological disorder that significantly impacts patients' lives and mental well‐being [1, 2]. The pathophysiology of CM involves both central and peripheral pain mechanisms, such as trigeminovascular system activation and the release of pro‐inflammatory mediators [3]. The current therapeutic landscape for migraine includes three classes of migraine‐specific drugs: triptans (5‐HT1B/1D receptor agonists), gepants (small‐molecule CGRP receptor antagonists), and CGRP monoclonal antibodies [4, 5]. Despite these pharmacological advances, which have significantly improved migraine management and provided relief for many patients, certain limitations persist, such as potential cardiovascular and hepatic effects [6]. Given these therapeutic challenges and migraine's complex pathophysiology, identifying new therapeutic targets is essential.

Furthermore, the trigeminal nucleus caudalis (TNC), a key hub in pain transmission, plays a central role in processing nociceptive information during the pathological progression of CM [7, 8, 9]. Our previous studies have shown that, under the CM background, the cellular composition and dynamic changes within the TNC are closely associated with the maintenance of central sensitization [10]. Meanwhile, the TNC, the first central site of the trigeminal nociceptive pathway, is responsible for processing sensory and pain signals from the peripheral orofacial area [11]. Increased neuronal excitability in the TNC is a key factor in central sensitization [12]. Importantly, microglia and astrocytes in the TNC have been reported as significant contributors to central sensitization, with their inhibition being a crucial mechanism for limiting inflammation and improving central sensitization [13] and astrocytes play critical roles in the modulation of pain within the TNC. Upon noxious stimulation or nerve injury, microglia in the TNC become activated and release pro‐inflammatory cytokines such as TNF‐α, IL‐1β, and IL‐6, contributing to neuronal hyperexcitability and the development of central sensitization [14]. Astrocytes also respond to persistent nociceptive input by undergoing reactive astrogliosis and releasing gliotransmitters such as glutamate, ATP, and inflammatory mediators, further amplifying pain transmission [15]. The bidirectional interactions between glial cells and neurons in the TNC are therefore essential for the initiation and maintenance of chronic orofacial pain conditions, including migraine, trigeminal neuralgia, and temporomandibular joint disorders [16].

Acupuncture, a traditional therapy with a long history in China, has been recognized by the World Health Organization as an effective treatment for a wide range of diseases [17]. According to a Cochrane review, acupuncture is shown to reduce migraine frequency and is at least as effective as prophylactic medication [18]. Building on clinical evidence showing that manual acupuncture is superior to non‐penetrating non‐acupoints [19], our previous research further verified its long‐term prophylactic benefits [20]. Although acupuncture has been shown to be an effective treatment for CM, its specific cellular mechanisms remain unclear. However, conventional bulk‐tissue analyses mask cell‐type‐specific dynamics. This hinders our understanding of the specific responses of different cell types to acupuncture intervention and obscures intercellular signaling pathways. Single‐cell RNA sequencing (scRNA‐seq) can delineate the cellular composition, heterogeneity, and gene expression profiles of tissues at single‐cell resolution [21], thereby revealing acupuncture's regulatory effects on specific cell types and the underlying molecular pathways. Previous studies have demonstrated that scRNA‐seq can successfully reveal differential therapeutic responses across distinct cell subpopulations and identify key molecular targets in pharmacological research on natural products [22], this provides a key insight for acupuncture research: as a traditional physical intervention, acupuncture may exert its therapeutic effects by modulating specific neural or immune cell subpopulations and their signaling networks.

In our previous research, we have clearly demonstrated the presence of metabolic‐immune reprogramming and dysregulated cell communication in glial cells in chronic migraine, which play a crucial role in the onset and maintenance of the condition [10]. Building on this finding, we aim to further investigate the cellular mechanisms underlying acupuncture treatment for chronic migraine using single‐cell RNA sequencing technology, with a particular focus on its potential to modulate specific neuronal or immune cell subpopulations and their signaling networks. Through in‐depth analysis of these cellular‐level changes, we hope to provide a more solid scientific foundation for the clinical application of acupuncture and offer theoretical support for the development of new therapeutic strategies in the future.

## 2 Materials and Methods

### 2.1 CM Rat Model

Given that the anatomical structure and physiological responses of male Sprague–Dawley (SD) rats more closely resemble human pathological conditions [23], 30 male SD rats (200 ± 20 g) were purchased from *dashuo* Animal Company in Chengdu (SCXK (chuan) 2025–0030) and housed under controlled conditions: temperature 25°C± 2°C, humidity 50% ± 10%, with a 12‐h light–dark cycle.

**FIGURE 1:**
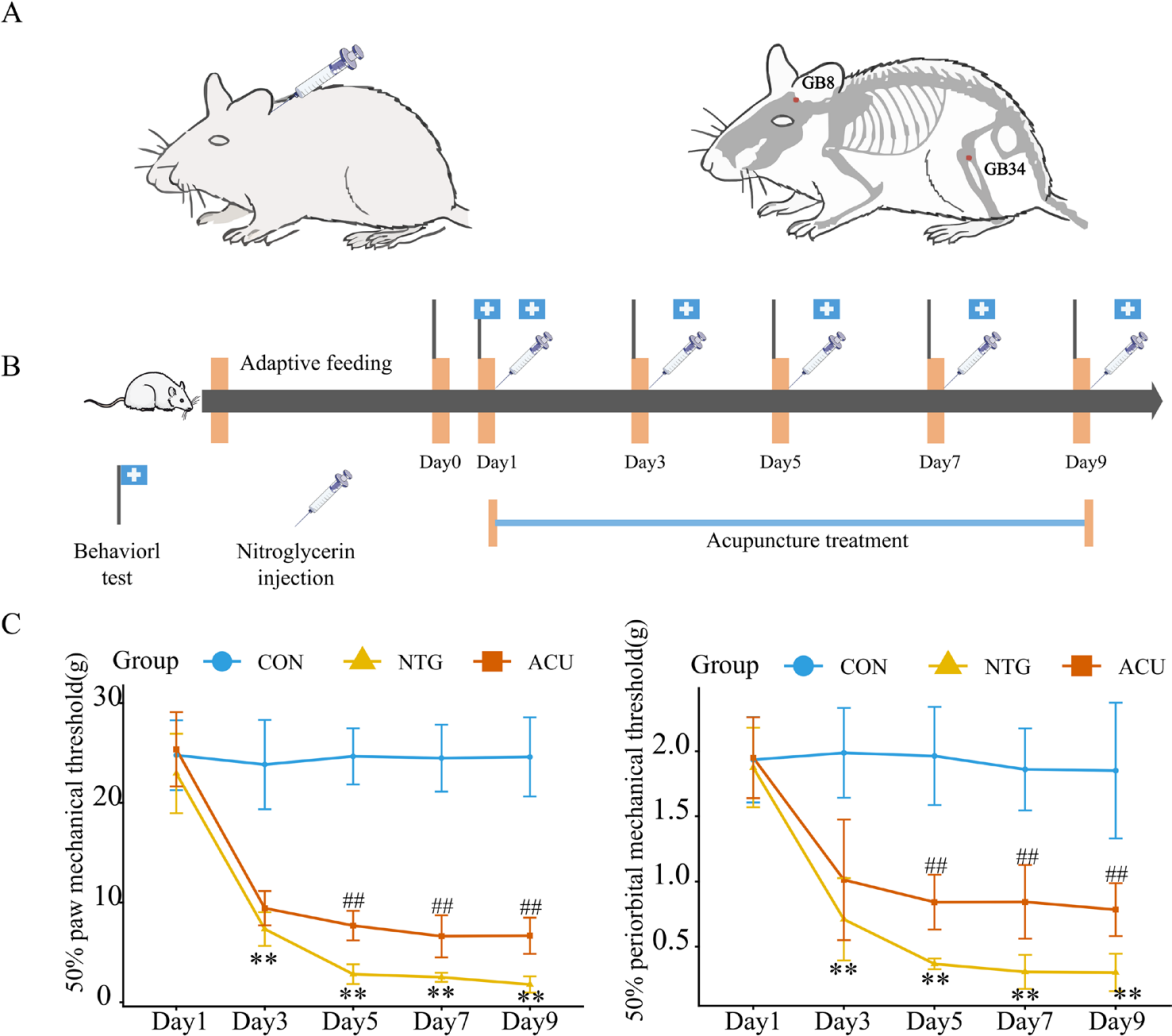
Acupuncture improves pain sensitization in NTG‐induced CM rats (A) Schematic representation of model induction. (B) Illustration of acupuncture points in rats. (C) Timeline of experimental procedures. (D, E) Acupuncture effectively inhibited the reduction of mechanical thresholds in both hind paw plantar and periorbital regions. Data are presented as mean ± SE. *p < 0.05 compared to CON group; ^#^ *p* < 0.05 compared to NTG group.

The CM model was established via subcutaneous injection of nitroglycerin (NTG, Yimin Pharmaceutical Co. Ltd., Beijing, China) [24]. Following a 7‐day acclimatization period, the animals were randomly divided into three groups (*n* = 10): Normal (CON), CM model (NTG), and acupuncture‐treated (ACU). Both NTG and ACU groups received NTG injections (10 mg/kg) on days 1, 3, 5, 7, and 9; the ACU group additionally underwent daily acupuncture treatment at the bilateral GB8 (Shuaigu) and GB34 (Yanglingquan) acupoints, for a total of 9 sessions (Figure 1A–C). The CON group received only saline injections. All the procedures were approved by the Experimental Animal Ethics Committee of Chengdu University of Traditional Chinese Medicine (Approval No. 2023DL‐047) [24].

### 2.2 Behavioral Assessment

Prior to the experiment, rats were acclimatized for 30 min in a quiet acrylic cage fitted with a wire mesh floor. The mechanical pain threshold was measured within 2 h before each treatment.

To determine the 50% paw mechanical threshold, Von Frey filaments with a force range of 1–26 g were applied vertically to stimulate the central area of the corresponding hind paw of each rat. A positive response was defined as paw withdrawal, limb trembling, or paw licking following stimulation.

For measuring the 50% periorbital mechanical threshold, rats were restrained in a holder with only their heads exposed to prevent movement. Von Frey filaments with a force range of 0.02–2 g were vertically applied to stimulate the rats' periorbital region. A positive response was identified as immediate head withdrawal or ipsilateral forelimb scratching of the facial area after stimulation.

### 2.3 Brain Tissue Dissociation and Preparation of Cell Suspensions

Data for the CON and NTG groups were derived from our prior publication [10], TNC tissues were freshly collected from three randomly selected rats in the ACU group. The tissues were mechanically dissociated with the Cell Nuclear Isolation Kit (Shbio, 52,009–10, Shanghai, China) following the manufacturer's protocol. Nuclei counts were determined using a cell counter (Thermo Fisher Scientific, Waltham, MA, USA). RNA barcoding and library preparation were performed with Chromium Single Cell 3′ Library (10X Genomics, Pleasanton, CA, USA) and Gel Bead Kit v3 (10X Genomics, Pleasanton, CA, USA) and sequenced on an Illumina NovaSeq 6000 platform (Illumina, San Diego, CA, USA).

### 2.4 Quality Control and Preprocessing of snRNA‐Seq Data

In our study, each biological replicate.i.e. Each rat (each rat) was sequenced independently, and each sample was prepared into a separate library with no pooling of samples. This ensured the independence of the data from each sample and avoided the potential for pseudo‐replication due to pooling.

For single‐cell RNA‐seq data processing, the Cell Ranger 3.0.1 pipeline was used to process the reads, employing the default and recommended parameters. Reads were aligned to the *Rattus norvegicus* reference genome (Rnor_6.0) using STAR. and gene‐barcode matrices were generated by counting unique molecular identifiers (UMIs). Cells with fewer than 500 or more than 4000 genes, mitochondrial gene content > 15%, or UMI/gene metrics deviating more than two standard deviations from the mean were excluded. Despite multiple washes after dissociation, ambient RNA from lysed cells can interfere with subsequent analysis [25]. Doublets were removed using DoubleFinder (version 2.0.3), and DecontX was used to estimate and remove cell contamination [26, 27]. Data normalization and scaling were performed using Seurat [28]. Highly variable genes (*n* = 2000) were selected using the FindVariableFeatures function. To address batch effects and ensure effective integration of data from different experimental batches, we applied the Harmony algorithm (version 1.2.0, https://github.com/immunogenomics/harmony) for batch effect correction. This method has been proven to achieve efficient integration of data from different batches, particularly when multiple batches are involved, by preserving biological signals while minimizing batch effects. In this study, after applying Harmony for batch effect correction, we were able to ensure that data from all groups (CON, NTG, and ACU) were compared within a unified analytical framework, thereby enhancing the reliability of data integration [29]. Finally, RunUMAP and FindClusters were used to identify and cluster cells.

### 2.5 Cell Type Identification

Cluster‐specific marker genes and differentially expressed genes (DEGs) across clusters were identified using the FindMarkers function, with a cutoff of min.pct = 0.25 to filter out genes detected in fewer than 25% of cells. The expression of marker genes in each cluster was compared to that of the entire cell population. Cell identities were assigned based on the CellMarker database (CellMarker 2.0, http://www.bio‐bigdata.center/index.html, accessed on 20 April 2025) [30]. To study the functional mechanisms of each cell cluster, subpopulations with sufficient cell numbers were extracted using the “SubsetData” strategy after preliminary annotation. These selected cells were then re‐clustered using t‐distributed stochastic neighbor embedding (t‐SNE), and annotated with the “FindClusters” function.

### 2.6 Differential Expression Analysis

The FindMarkers function in Seurat was employed to identify DEGs using default parameters. Specifically, comparisons were made between the NTG and CON groups, as well as between NTG and ACU. DEGs were defined as those with an adjusted *p* < 0.05 and an absolute log‐fold change (|logFC|) > 0.25. Gene Ontology (GO) and the Kyoto Encyclopedia of Genes and Genomes (KEGG) pathway were performed using clusterProfiler (v4.8.3) with gene sets from the msigdbr (v7.5.1) database. Terms with *p* < 0.05 were considered significantly enriched. The results were visualized using ggplot2 and pheatmap [31].

### 2.7 Functional Enrichment Analysis

In this study, gene set variation analysis (GSVA) was performed using the msigdbr package to identify activated functional pathways within different cell clusters. Pathways with corrected *p*‐values (*p* > 0.05) were excluded from further analysis. Gene set enrichment analysis (GSEA) using the clusterProfiler R package was also performed to investigate differential pathways between cell subpopulations. The AUCell package (v1.22.0) was utilized to compute gene expression feature scores to assess functional differences between different cell populations [32].

### 2.8 Single‐Cell Trajectory Analysis

To characterize potential differentiation processes between cells, various analysis methods including CytoTRACE (version 0.3.3, https://cytotrace.stanford.edu/) and Monocle2 (version 2.28.0) were employed for single‐cell differentiation trajectory analysis [33]. CytoTRACE was used to assess cell differentiation, where higher CytoTRACE scores indicated lower differentiation levels and vice versa. Briefly, a subset of the raw data of the target cell cluster is first extracted and the expression difference of each gene between cells is calculated using a scatter table. Variable genes are then identified based on the mean expression level (parameters “mean_express > = 0.1” and “dispersion_empirical > = 1 * dispersion_fit”), thus defining the developmental process. The dimensionality of the data was subsequently reduced to two components using the “DDRTree” method, and the trajectory was inferred with the “orderCells” function. The ‘BEAM’ function was employed to compute genes that are specifically expressed along each branch of the trajectory. For visualizing the branch‐specific expression patterns, we utilized the ‘plot_genes_branched_heatmap’ function implemented in Monocle, considering only those genes with a q‐value less than 1e‐4 as the input for the heatmap. Subsequently, gene clusters were further delineated using k‐means clustering. To explore the functional roles of genes within each cluster, we conducted gene ontology analysis with the clusterProfiler R package.

### 2.9 Cell–Cell Communication Analysis

Inter‐cellular communication was analyzed using the R CellChat package (https://github.com/sqjin/CellChat) [34], which is currently limited to human and mouse genomes, the rat genes were converted to their mouse orthologs using the homologene R package (v1.4.68.19.3.27). Leveraging a precompiled mouse PPI network from the Secreted Signaling Database for pathway inference, we performed cell–cell communication analysis using core functions to estimate communication probabilities, reconstruct the signaling network, and identify differentially expressed interactions between groups. Therefore, the current analysis results should be considered as speculative results derived from homologous gene mapping, and should be interpreted with caution.

### 2.10 Statistical Analysis

Statistical analysis between two groups was performed using an unpaired *t*‐test. Behavioral results were analyzed using a two‐way repeated‐measures ANOVA with a Bonferroni post hoc test. A *p* < 0.05 was chosen to determine statistical significance.

## 3 Results

### 3.1 Acupuncture Improves Pain‐Related Behavior in NTG‐Induced CM Rats

Before the model establishment, baseline data showed no significant differences in mechanical pain among the three groups of rats. After 9 days of intermittent NTG administration, mechanical pain significantly decreased in the NTG group compared to the CON group receiving saline (*p* < 0.05). The trend of mechanical pain decline was alleviated after acupuncture treatment (Figure 1D,F). These results confirm that the CM model we established successfully induced pain sensitization in rats, and acupuncture treatment significantly improved pain behaviors in the rats [35].

### 3.2 Characterization of TNC Cell Types

**FIGURE 2:**
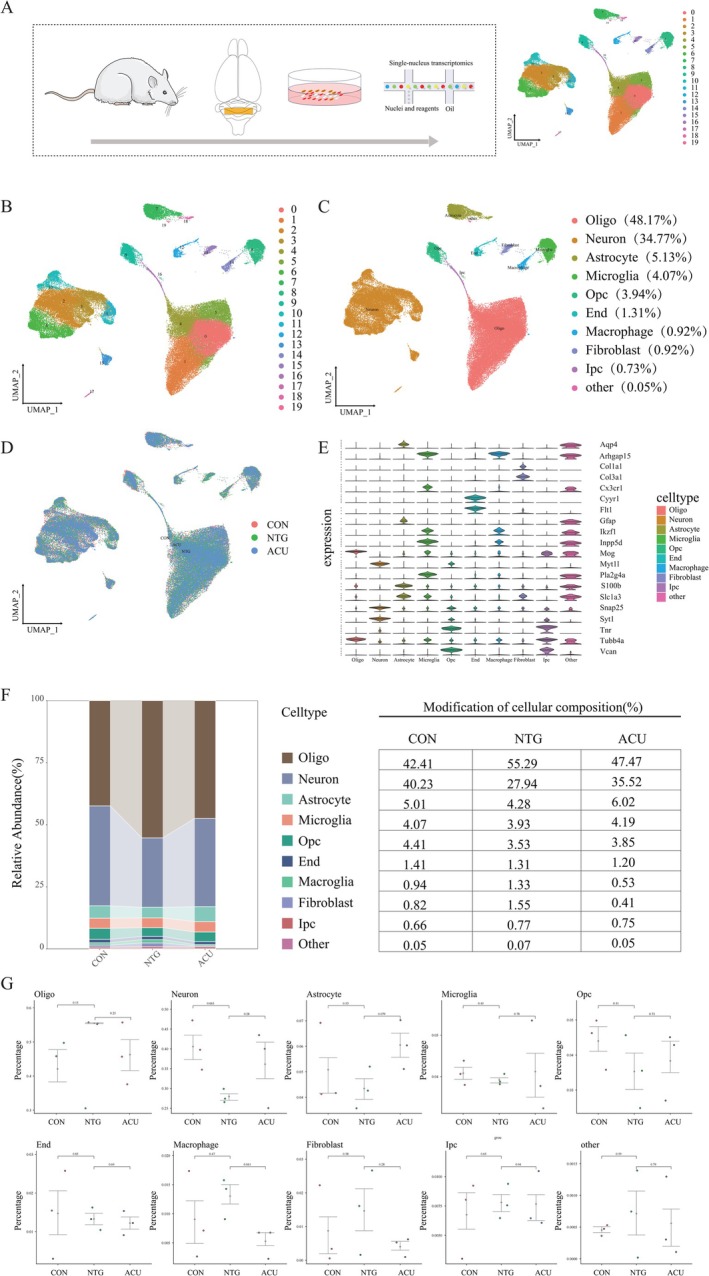
Experimental pipeline and single cell landscape of acupuncture treatment in CM rats. (A) Experimental scheme and workflow diagram in the study. (B and C) Cell types of sequencing cells projecting on UMAP visualization. Oligo, oligodendrocytes; Opc, oligodendrocyte precursor cells; End, endothelial cells; Ipc, oligodendrocyte intermediate precursor cells. The percentages in the brackets represent the percentage of each cell type. (D) UMAP visualization showing the group attribute of cells. (E) Violin plot displaying expression of classical marker in responding cell type. The Y‐axis shows the logscale normalized reads count. (F) Bar plot depicting cellular composition in the three groups (CON, NTG and ACU). The table beside the bar plot contains the specific percentages. (G) Statistical analysis of cellular changes in cell types within CON, NTG and ACU.

To assess the effects of acupuncture on CM, we performed snRNA‐seq on frozen TNC tissue from the ACU (three cohorts) combined with data for the CON and NTG groups derived from our prior publication. The experimental workflow is outlined in Figure 2A. After quality control, a total of 82,513 cells were obtained (28,447 from the CON group, 25,812 from the NTG group, and 28,254 from the ACU group). The data were reduced in dimension (resolution 0.5) and visualized using UMAP (Figure 2B–E). Based on classical cell‐type marker genes, we successfully identified nine cell lineages: oligodendrocytes (Oligo, 48.17%, high expression of Tubb4a and Mog), neurons (Snap25/Syt1/Myt1), astrocytes (S100b/Slc1a3/Aqp4/Gfap), microglia (C×3cr1/Pla2g4a/Inpp5d), oligodendrocyte precursor cells (Opc, Tnr/Vcan), endothelial cells (End, Flt1/Cyyr1), and macrophages (Arhgap15/Ikzf1), fibroblasts (Col3a1/Col1a1). In addition, we annotated an intermediate progenitor population (Ipc), which transcriptionally bridged proliferative OPCs and terminally differentiated oligodendrocytes, consistent with previous single‐cell studies [36]. Clusters of cells that co‐expressed microglial and astrocyte‐specific genes were classified as an unknown cell type. UMAP visualization of marker gene expression (Figure S1) further confirmed cell type specificity.

Notably, compared to the CON group, the proportion of oligodendrocytes in the NTG group significantly increased from 42.41% to 55.29%, while macrophages rose from 0.94% to 1.33%. In contrast, the proportions of neurons, astrocytes, microglia, and OPCs all decreased. Acupuncture intervention partially reversed these abnormal cell proportions (Figure 2F,G), suggesting that the TNC cell composition disorder induced by nitroglycerin can be improved by acupuncture treatment.

### 3.3 Cellular Heterogeneity in Response to Acupuncture Treatment

To clarify the molecular mechanisms regulated by acupuncture, DEGs for each cell type were identified using the FindMarkers function in Seurat. By comparing the distribution of upregulated and downregulated DEGs between the NTG and CON groups, as well as the ACU and NTG groups (Figure S2A,B), we observed significant changes in the DEG expression profiles of neurons, oligodendrocyte precursor cells (Opc), astrocytes, and oligodendrocytes (Oligo) (Figure S2C–G). Notably, neurons exhibited the most pronounced DEG changes following acupuncture treatment (Figure S2H), followed by astrocytes and microglia, with the majority of DEGs displaying cell‐type‐specific expression patterns, suggesting that acupuncture intervention triggers a cellular heterogeneity response.

**FIGURE 3:**
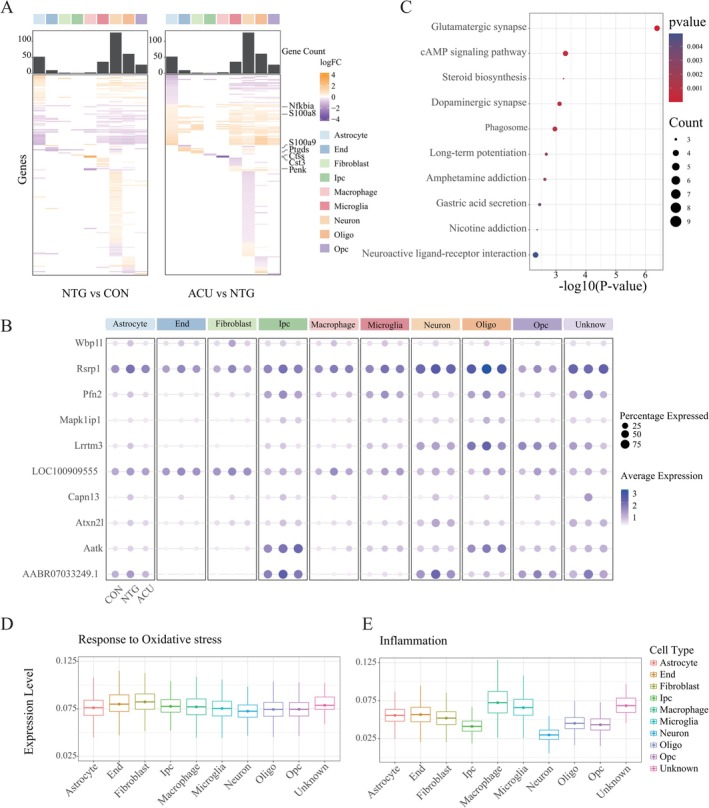
Analysis of differential gene expression and cellular heterogeneity response to acupuncture treatment. (A) Heatmap of log2 (fold change) of rescuing genes in nine cell types (left: NTG vs. CON; right: ACU vs. NTG). Gene symbols beside heatmap representing genes related to inflammation and oxidative stress. (B) Expression levels of up‐regulating genes in the NTG group, which display significant variability among cell types. (C) Dot plot displaying pathway enrichment of rescuing genes. (D) Distribution of response to oxidative stress module score in identifying cell types. (E) Distribution of inflammation module score in identifying cell types.

Further analysis revealed that acupuncture specifically rescued the expression of several genes with aberrant regulation in neurons, astrocytes, and microglia (e.g., Nfkbia, S100a8, S100a9, and Penk), which are primarily enriched in pathways related to oxidative stress and inflammation (Figure 3A,B). Among these, the cAMP signaling pathway and the phagosome pathway were particularly prominent (Figure 3C). Functional module analysis showed that oxidative stress responses did not exhibit cell‐type differences, whereas inflammation scores were significantly elevated in microglia, macrophages, and astrocytes (Figure 3D,E) [37].

In conclusion, acupuncture exerts its therapeutic effects by modulating the expression of inflammation‐ and oxidative stress‐related genes in specific cell types (neurons, microglia, and astrocytes). To further investigate the underlying mechanisms, focused analyses will be conducted on these three cell types in the subsequent sections.

### 3.4 Acupuncture Intervention Alleviates Nitroglycerin‐Induced Neuronal Injury

Neurons, as the central executors of neural function, are closely implicated in the complex pathophysiology of CM [38]. Given that neurons exhibited the most significant gene rescue effects following acupuncture treatment, we performed an in‐depth analysis of their expression profiles. Hierarchical clustering revealed that the neuronal gene expression pattern in the ACU group was highly similar to that of the CON group, whereas significantly deviating from the NTG group (Figure S3A,B), suggesting that acupuncture can reverse nitroglycerin‐induced neuronal injury. A Venn diagram identified 59 acupuncture‐specific regulated overlapping DEGs, which were subsequently used to construct a protein–protein interaction (PPI) network via the STRING database. Using the maximum clique centrality (MCC) criterion, the top 10 core target genes were identified (Figure S3C,D), which include key molecules involved in regulating synaptic plasticity and energy metabolism.

GSEA pathway enrichment further revealed that, in the NTG group, synaptic transmission‐related pathways (calcium signaling, glutamatergic synapses, long‐term potentiation) were significantly activated, whereas oxidative phosphorylation and ATP synthesis pathways were notably suppressed. Acupuncture intervention reversed these abnormalities (Figure S3E–H), suggesting that acupuncture protects neurons by restoring the “excitability‐metabolism” homeostasis. CellChat analysis (Figure 6A–C) indicated that the abnormal communication patterns between microglia, astrocytes, and neurons in the NTG group were normalized following acupuncture. Together with the discovery of numerous “rescue genes” in astrocytes and microglia, these findings suggest that glial cells may participate in the neurorepair process induced by acupuncture by modulating the neuronal microenvironment.

### 3.5 Acupuncture Treatment Alleviates Nitroglycerin‐Induced Microglial Injury

Microglia, as the immune surveillance cells of the central nervous system, play a pivotal role in regulating neuroinflammation and modulating neuronal susceptibility [39]. Enrichment analysis of DEGs in microglia from the NTG group revealed that their aberrant activation was primarily associated with imbalances in tissue homeostasis (e.g., tissue homeostasis, anatomical structure homeostasis) and apoptosis/phagocytosis‐related pathways (Figure S4A). Differential expression profile analysis further demonstrated that microglia in the NTG group exhibited a prominent pro‐inflammatory phenotype: the pro‐inflammatory marker tumor necrosis factor‐alpha (TNF‐α) and M1‐type marker CD86 were upregulated, while tissue repair‐related molecules such as Arg1 and M2‐type marker CD163 were downregulated (Figure 5B). Acupuncture intervention effectively reversed these trends, suggesting that it modulates the polarization state of microglia (M1/M2 phenotype shift) to balance inflammation and repair functions [40].

Based on transcriptional heterogeneity analysis, microglia were classified into five functional subgroups (M1–M5). Compared to the CON group, the NTG group exhibited a significant increase in the pro‐inflammatory M1/M5 subgroups (Figure S4C,E) and a decrease in the anti‐inflammatory/repair M2/M4 subgroups. Acupuncture intervention specifically reshaped the distribution of subgroups, inhibiting the expansion of pro‐inflammatory subtypes and promoting the proliferation of repair subtypes. Functional scores (AUcell inflammation score) and pathway enrichment analysis (GSVA) showed that M2/M4 subgroups were enriched in metabolic regulation (PPAR signaling, fatty acid metabolism) and anti‐inflammatory pathways (autophagy regulation), while M1/M5 subgroups were enriched in pro‐inflammatory pathways such as Toll‐like receptors (TLRs) and NOD‐like receptors (Figure S4D,F), indicating that acupuncture improves the neuroinflammatory microenvironment by modulating the balance of microglial subtypes.

**FIGURE 4:**
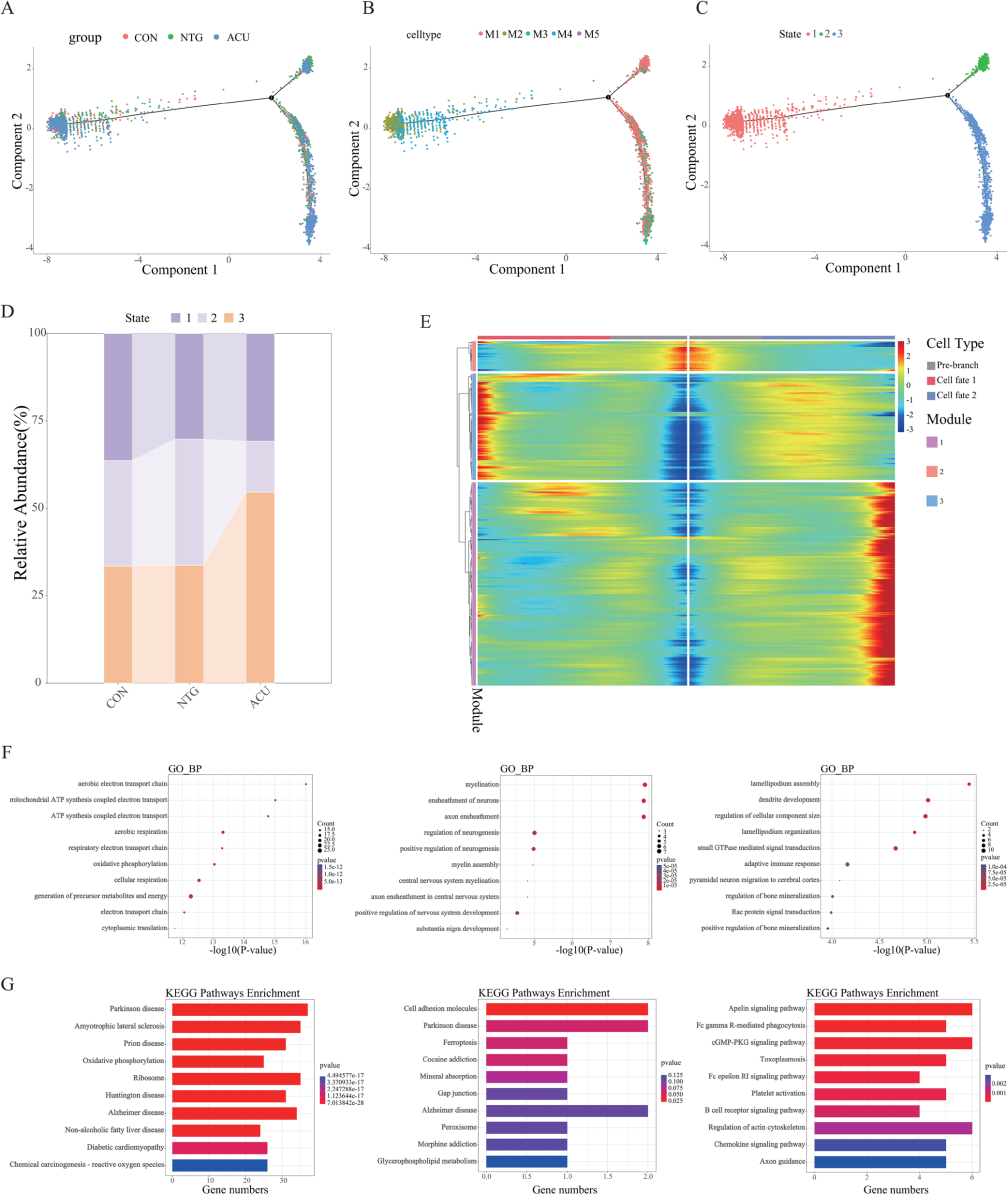
Differentiation trajectories of microglia subtypes. (A–C) Pseudo‐time trajectory of microglia with different groups, subtypes, and states. (D) Proportion of all the microglia in different states. (E) Pseudo‐time ordered heatmap of three differentially expressed gene modules between two obvious fates at branch point one. (F) GO enrichment analysis of different gene modules (left to right: Module1 to Module3). (G) KEGG enrichment analysis of different gene modules (left to right: Module1 to Module3).

To further elucidate the dynamic evolution of microglia in response to acupuncture, pseudotime analysis constructed three distinct fate trajectories (Figure 4A–C). Following NTG intervention, microglial cells shifted toward stage 2 (pro‐inflammatory state), while acupuncture reprogrammed them toward stage 3 (repair state) (Figure 4D). Gene expression module analysis revealed that fate 1 (stage 1 → 2) was associated with cell migration (lamellipodium assembly, dendritic development) and phagocytosis‐related pathways (FcγR‐mediated phagocytosis, cGMP‐PKG signaling); fate 2 (stage 1 → 3) was closely linked to energy metabolism (oxidative phosphorylation, mitochondrial ATP synthesis) and neuroprotection (Parkinson's disease pathway) (Figure 4E–G). These results suggest that acupuncture reshapes the developmental trajectory of microglia, coordinating their inflammatory response and metabolic adaptation, thereby exerting neuroprotective effects.

### 3.6 Acupuncture Intervention Improves Nitroglycerin‐Induced Dysfunction of Astrocytes

Astrocytes, as central regulators of brain homeostasis, play a critical role in the pathogenesis of CM when their function becomes disrupted [41]. Enrichment analysis of DEGs in astrocytes from the NTG group revealed that their dysfunction primarily involved synaptic transmission regulation (e.g., glutamatergic synaptic pathways) and neuronal development‐related pathways (e.g., positive regulation of neuronal projection development) (Figure S5A). The differential expression profile showed that key genes involved in glutamate metabolism (Glul, Slc1a3) and the A2‐type astrocyte marker S100b were significantly downregulated in the NTG group, whereas acupuncture intervention effectively restored their expression levels (Figure S5B). These results suggest that acupuncture may improve the neuronal microenvironment by reshaping glutamate homeostasis [42].

Based on transcriptional heterogeneity analysis, astrocytes were classified into four functional subgroups (A1‐A4). Compared to the CON group, the NTG group exhibited a significant increase in the pro‐inflammatory A1/A3 subgroups and a decrease in the repair‐related A2 subgroup, with the A4 subgroup completely absent. Acupuncture intervention reversed this trend, inhibiting the expansion of pro‐inflammatory subtypes and inducing the regeneration of repair‐related A2/A4 subgroups (Figure S5C,D). GSVA pathway enrichment analysis revealed that the A1/A2 subgroups were highly enriched in inflammation‐related pathways (TNF signaling, IL‐17 signaling), while A4 subgroups specifically activated repair‐related pathways (TGF‐β signaling, Wnt signaling). The A1 subgroup was also enriched in fatty acid metabolism, while the A2 subgroup was associated with oxidative phosphorylation and glutathione metabolism (Figure S5E). These findings suggest that acupuncture regulates the balance of subgroups, coordinating the suppression of inflammation with metabolic repair functions.

**FIGURE 5:**
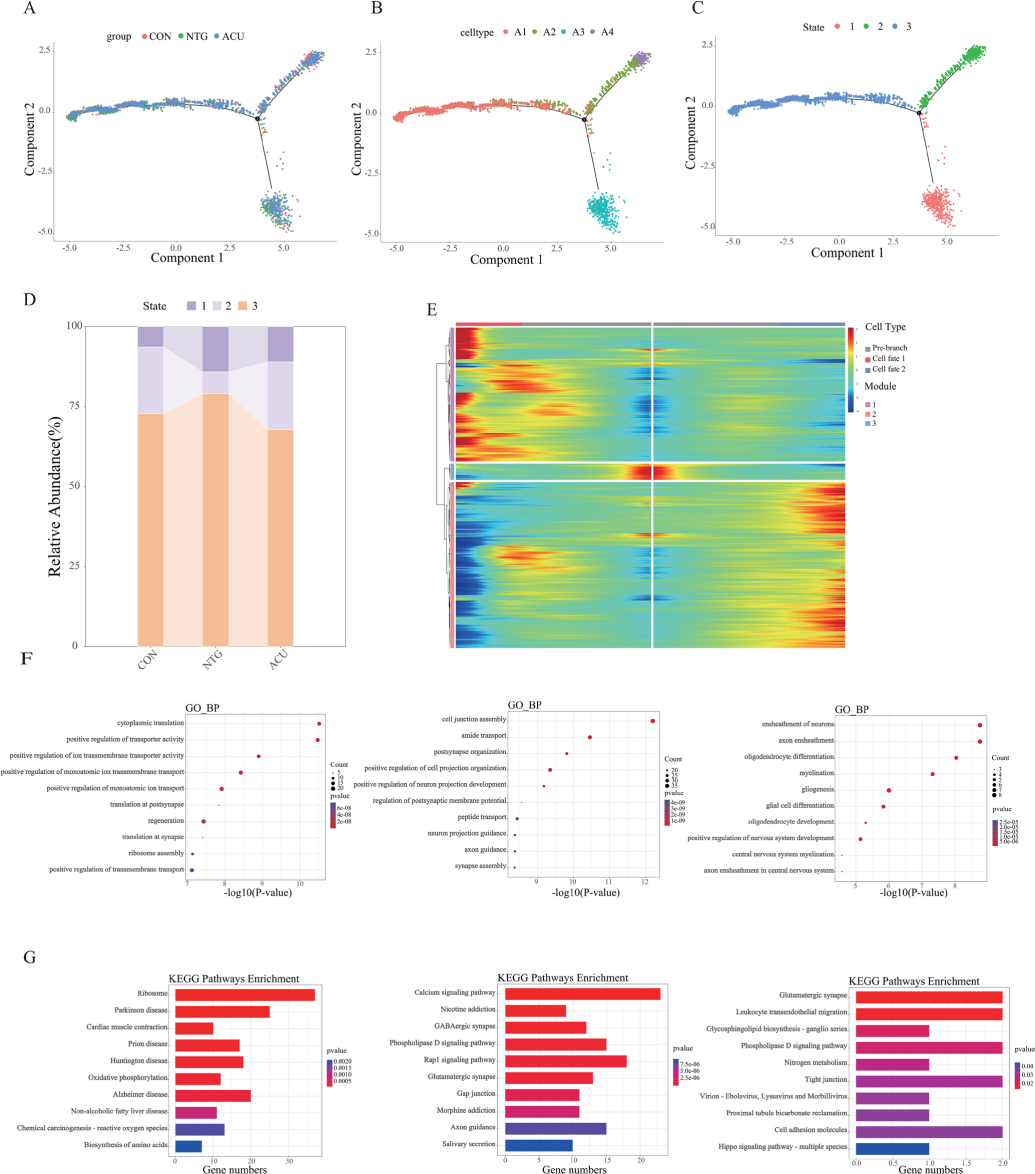
Differentiation trajectories of astrocyte subtypes. (A‐C) Pseudo‐time trajectory of astrocytes with different groups, subtypes, and states. (D) Proportion of all the astrocytes in different states. (E) Pseudo‐time ordered heatmap of three differentially expressed gene modules between two obvious fates at branch point one. (F) GO enrichment analysis of different gene modules (left to right: Module1 to module3). (G) KEGG enrichment analysis of different gene modules (left to right: Module1 to module3).

To explore the dynamic characteristics of astrocyte state transitions, pseudotime analysis was used to construct two developmental trajectories (Figure 5A–C). After NTG intervention, astrocytes shifted toward stage 3 (functional suppression state), whereas acupuncture reprogrammed them toward stage 2 (activation state) (Figure 5D). Gene module analysis revealed that fate 1 (stage 1 → 2) was associated with the activation of calcium signaling and GABAergic synaptic genes, while fate 2 (stage 1 → 3) was linked to ribosomal function and Parkinson's disease pathways (Figure 5E–G). These results suggest that acupuncture promotes the recovery of astrocyte function by enhancing synaptic regulation and energy metabolism.

### 3.7 Acupuncture's Effect on Glial Communication Networks Inferred Through Homologous Gene Mapping

**FIGURE 6:**
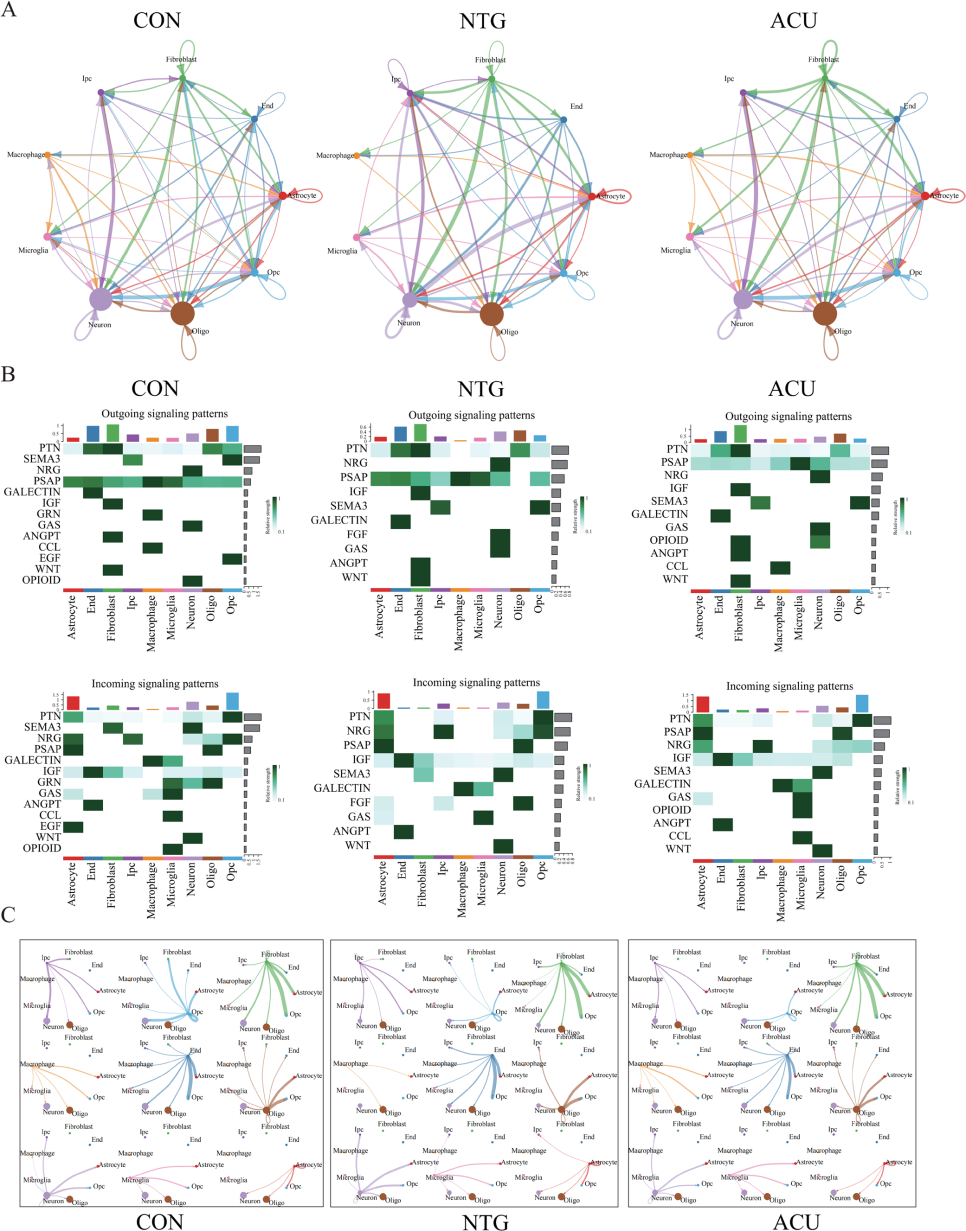
ACU mediated intercellular crosstalk in TNC with CM. Overall networks of intercellular crosstalk within cell types of CON, NTG, and ACU groups. (B) Ligand‐receptor interactions among different cell types in CON, NTG, and ACU groups. (C) Specific intercellular interactions among different cell types in CON, NTG, and ACU groups.

To investigate how ACU modulates intercellular signaling in the TNC during CM, we employed CellChat to infer ligand‐receptor interactions among cell populations. The speculative results inferred through homologous gene mapping suggest that, compared with the control group, NTG‐treated samples exhibited alterations in intercellular communication to varying degrees. ACU intervention partially modulated these alterations; notably, while NTG increased interactions between microglia and Ipc cells, ACU treatment reduced this change (Figure 6A,C). The dissection of outgoing and incoming signaling profiles revealed that several canonical communication families, including OPIOID, CCL, and EGF, were broadly diminished in the NTG group. Notably, only FGF signaling was consistently upregulated as incoming signals across multiple cell types, particularly in Oligo. ACU intervention increased the NTG‐suppressed OPIOID and CCL signaling profiles while reducing the enhanced FGF signaling profiles.

## 4 Discussion

This study used an NTG‐induced CM rat model to explore the therapeutic effects of acupuncture on pain sensitization and neuronal dysfunction. Through snRNA‐seq, we identified profound changes within the TNC region, including remodeling of cellular composition, reprogramming of gene expression patterns, and reorganization of intercellular communication networks. These findings offer a novel perspective on acupuncture's mechanisms by revealing multi‐cellular and network‐level pathways through which acupuncture alleviates central sensitization.

In the behavioral assays, following NTG treatment, rats in the model group exhibited a significant decrease in mechanical pain thresholds. However, acupuncture‐treated rats maintained a significantly higher pain threshold, suggesting that acupuncture effectively alleviates pain sensitization. This observation is consistent with previous studies that emphasize the central role of inflammation in the pathogenesis and maintenance of chronic pain [43]. NTG‐induced pain sensitization is sustained through central nervous system inflammation [44]. Based on these results, we hypothesize that acupuncture may modulate the interactions between immune and neural cells, thereby alleviating local inflammatory states and reducing pain.

Single‐nucleus transcriptomic analysis further revealed that NTG treatment significantly disrupted the cellular composition within the TNC, particularly altering the proportions of immune cells. Acupuncture partially restored the proportions of certain cell types, especially glial cells, suggesting that acupuncture may help modulate the local immune environment, thereby alleviating immune dysfunction associated with chronic pain. Literature has highlighted the critical role of glial cells, particularly microglia and astrocytes, in the maintenance of chronic pain [10]. Our findings provide novel cellular‐level evidence supporting the role of acupuncture in modulating glial activity and immune responses.

Although previous studies have reported that acupuncture can reduce the levels of inflammatory mediators [45, 46], our single‐cell data provide new evidence supporting this finding by demonstrating that acupuncture significantly regulates the expression of genes associated with inflammation and oxidative stress—such as Nfkbia, S100a8, S100a9, and Penk—in neurons, astrocytes, and microglia. Furthermore, acupuncture activates the cAMP signaling pathway and phagocytosis mechanisms, effectively reversing the aberrantly activated state. This mechanism is consistent with literature discussing the role of oxidative stress pathways in neural recovery [47, 48]. In addition, based on the premise that synaptic hyperexcitability and disrupted energy metabolism are key factors in the maintenance of pain sensitization [49, 50], our findings show that acupuncture significantly restores synaptic transmission‐related pathways (e.g., calcium signaling, glutamatergic transmission, long‐term potentiation), while inhibiting oxidative phosphorylation and ATP synthesis pathways. This aligns with recent views on the critical role of excitability and metabolic balance in maintaining neural homeostasis [51, 52], providing new insights into acupuncture's neuroprotective mechanisms.

On the glial cell front, previous studies have shown that glial cell dysfunction in chronic pain conditions is a critical factor in pain persistence [53]. On the glial cell front, previous studies have shown that glial cell dysfunction in chronic pain conditions is a critical factor in pain persistence [54]. In our study, we observed that in the model group, microglia displayed a pro‐inflammatory phenotype, with increased expression levels of TNF‐α and CD86, which is consistent with previous reports [55]. Acupuncture not only promoted the phenotypic shift of microglia toward a reparative state but also restored the expression of glutamate metabolism markers and S100b in astrocytes, highlighting the multifaceted role of acupuncture in improving the local inflammatory environment. Moreover, glial remodeling in CM leads to the reorganization of cell–cell communication networks. Our intercellular communication analysis provides new insights into how acupuncture may alleviate central sensitization in CM. We observed widespread suppression of key neuroimmune signaling pathways, such as OPIOID and CCL, in the NTG model, which suggests impaired homeostatic communication within the TNC. The OPIOID pathway, known for its endogenous pain‐inhibitory function [56], and the CCL pathways, involved in modulating neuroimmune activation [57], are critical for maintaining pain thresholds. Their reduction suggests a failure of endogenous analgesic and immunoregulatory mechanisms in CM. In contrast, the specific upregulation of FGF signaling, particularly its influx into oligodendrocytes, may indicate a compensatory response related to neuronal stress or a maladaptive process contributing to central sensitization. This aligns with the known roles of FGF in synaptic plasticity and glial reactivity [58]. The restorative effects of acupuncture are particularly notable, as it reverses NTG‐induced suppression of OPIOID and CCL signaling, promoting recovery of endogenous pain control and immune homeostasis. Furthermore, the specific reduction of aberrant microglia‐Ipc interactions and normalization of FGF signaling by acupuncture suggests a targeted effect on non‐neuronal cells, indicating that acupuncture's therapeutic effects extend beyond neurons to regulate the broader glial‐neuronal network involved in pain processing.

Overall, through multi‐level data analysis, this study validates the significant efficacy of acupuncture in alleviating pain sensitization in NTG‐induced CM rats. Our findings elucidate its mechanisms of action from the perspectives of cellular composition, signaling pathways, and intercellular communication. By modulating the TNC neuroimmune network, reshaping cellular composition, and regulating gene expression, acupuncture alleviates neuroinflammation and improves CM pathology, offering a new single‐cell‐level perspective on its therapeutic efficacy.

### 4.1 Limitations

While this study offers valuable insights into the molecular mechanisms of acupuncture, several limitations must be acknowledged. First, the rat model used does not fully replicate human CM pathophysiology, and species‐specific differences may influence acupuncture's effects. Additionally, while snRNA‐seq provides important cell‐specific data, it lacks spatial information, limiting our understanding of the specific anatomical regions in the TNC impacted by acupuncture. Future studies should utilize spatial transcriptomics to map acupuncture's brain‐wide effects more comprehensively. Despite rigorous efforts to minimize batch effects, subtle differences between experimental batches could still affect data comparability and result accuracy. Future work should include larger sample sizes and multi‐batch validation to further assess and reduce batch effects. Finally, while using male rats helps avoid confounding hormonal fluctuations seen in females, this choice may limit the broader applicability of the findings. Future studies should include more diverse samples to validate these results and ensure their relevance to a wider patient population.

## 5 Conclusion

In summary, this study sheds light on the cellular and molecular mechanisms through which acupuncture alleviates CM, providing novel insights into its effects on oxidative stress, inflammation, and neuronal function in the TNC. These findings offer important implications for both basic science and clinical practice, supporting the potential of acupuncture as a non‐invasive, adjunctive therapy for migraine treatment.

## Author Contributions

Shiqi Sun: Writing – original draft, Visualization, Software, Resources, Project administration, Methodology, Investigation, Formal analysis, Data curation, Conceptualization. Shuangyuan Hu and Mingsheng Sun: Writing – original draft, Visualization, Software, Resources, Methodology, Formal analysis, onceptualization. Zili Tang: Writing – original draft, Visualization, Software, Formal analysis, Conceptualization. Yuyan Wang, Longyao Xu, and Puhua Cao: Writing – original draft, Conceptualization. Lu Liu: Writing – original draft, Validation, Formal analysis. Jing Yuan, Rong Wang, and Ying Chen: Writing – original draft, Visualization, Formal analysis, Data curation. Ling Zhao: Writing – review and editing, Supervision, Project administration, Methodology, Conceptualization, Funding acquisition.

## Funding

This study was supported by the National Natural Science Foundation of China under grant numbers 82274664 and 82430124.

## Ethics Statement

This study was approved by the Experimental Animal Ethics Committee of Chengdu University of Traditional Chinese Medicine (2023DL‐047).

## Consent

All authors have reviewed and approved the final manuscript and consent to its publication.

## Conflicts of Interest

The authors declare no conflicts of interest.

## Supporting information


**Figure S1:** The expression of selected marker genes in different clusters of brain cells.

**Figure S2:** Differential gene expression analysis and statistical assessment in each cell type.
(A) Volcano plot displaying the distribution of differentially expressed genes in NTG vs. CON. (B) Volcano plot displaying differential expressed genes in ACU vs. NTG. (C) Expression of top 3 up‐regulating genes in NTG vs. CON in each cell type. (D)
Expression of top 3 down‐regulating genes in NTG vs. CON in each cell type. (E) Expression of top 3 up‐regulating genes in ACU vs. NTG in each cell type. (F) Expression of top 3 down‐regulating genes in ACU vs. NTG in each cell type. (G) Statistics of differentially expressed genes in NTG vs. CON. The top panel representing number of DEGs induced during NTG basing on cell type; the bottom panel showing number of genes detected in only one cell type versus those detected in multiple cell types. (H) Statistics of differentially expressed genes in ACU vs. NTG. The top panel representing number of DEGs induced during NTG basing on cell type; the bottom panel showing number of genes detected in only one cell type versus those detected in multiple cell types.

**Figure S3:** Acupuncture treatment relieves neuron injury induced by CM.
(A) Volcano plots showing the DEGs of neurons between the ACU and CON group. (B) Venn diagram of the DEGs of neurons in various groups. (C) Venn diagram of the top 100 DEGs in neurons from different groups. (D) Top ten hub genes identified using the cytoHubba from Cytoscape based on MCC scores. (E‐F) GSEA enrichment analysis of neurons, E: NTG vs. CON (GO enrichment); F: ACU vs. NTG (GO enrichment). (G‐H) GSEA enrichment analysis of neurons, G: NTG vs. CON (KEGG enrichment); H: ACU vs. NTG (KEGG enrichment).

**Figure S4:** Acupuncture treatment relieves Microglial injury induced by CM.
(A) Function enrichment of DEGs in Microglia (NTG vs. CON, left: GO enrichment; right: KEGG enrichment). (B) Heatmap of average expression of specific expressed genes in each group. (C) Re‐clustering of Microglial (dividing into M1‐M5, and Low_quality). (D) Distribution of inflammation module score in each subtype. (E) Cellular composition of six subtypes in CON, NTG, and ACU. (F) GSVA analysis of different microglia subclusters.

**Figure S5:** Acupuncture treatment relieves Astrocytes injury induced by CM.
(A) Function enrichment of DEGs in Astrocytes (NTG vs. CON, left: GO enrichment; right: KEGG enrichment). (B) Heatmap of average expression of specific expressed genes in each group. (C) Re‐clustering of Astrocytes (dividing into A1‐A4, and Low_quality). (D) Cellular composition of six subtypes in CON, NTG, and ACU. (E) GSVA analysis of different Astrocytes subclusters.

## Data Availability

The data that support the findings of this study are available on request from the corresponding author. The data are not publicly available due to privacy or ethical restrictions.
