# Supplementary figures and images for: Single‐Nucleus RNA‐Seq Reveals Neuroprotective Effects of Acupuncture in Chronic Migraine Through Modulation of Glial Subtypes
Source: CNS Neurosci Ther. 2026 Jun 16;32(6):e70981. doi: 10.1002/cns.70981 (PMC13270986; doi:10.1002/cns.70981)

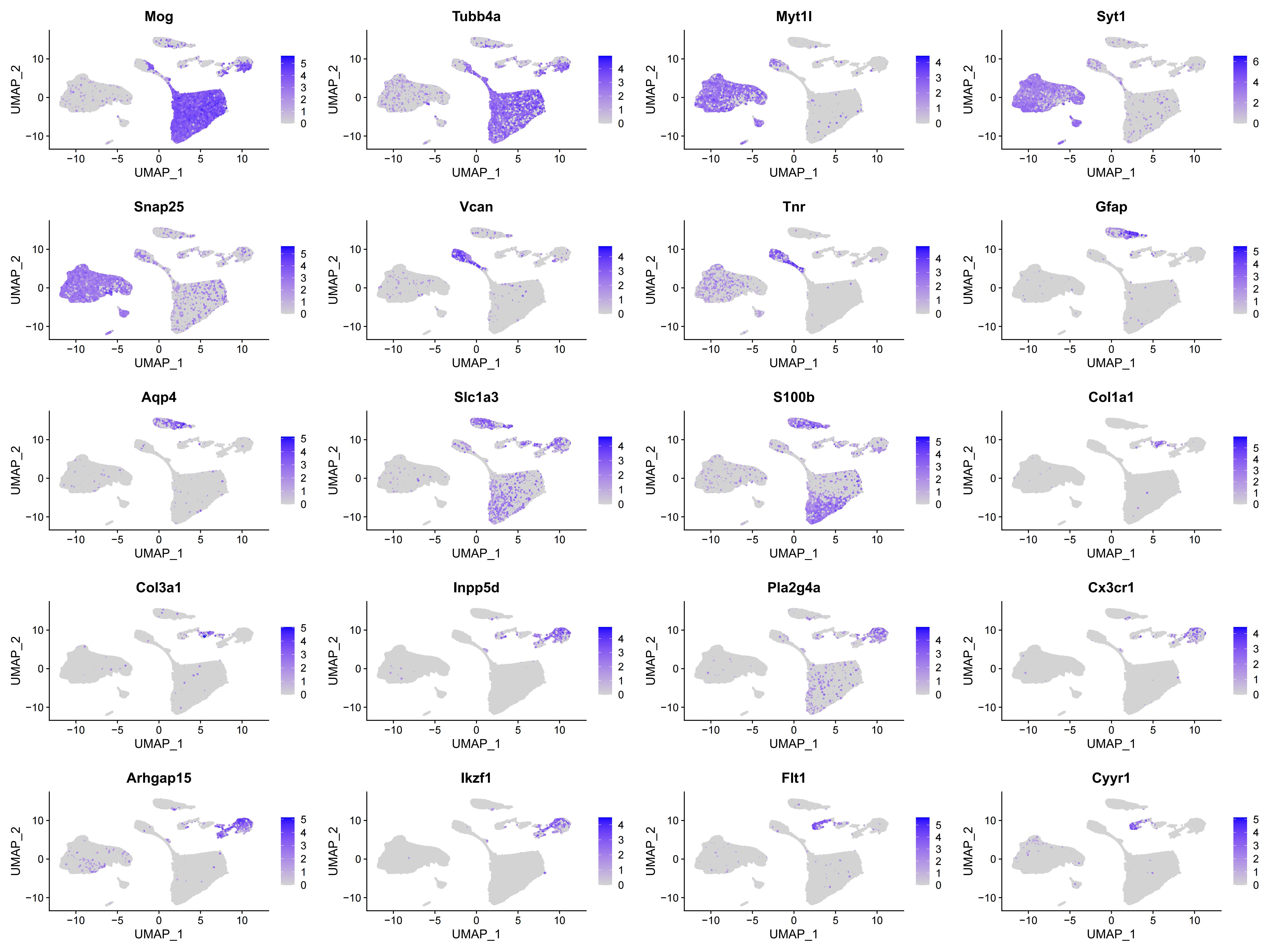

Supplement: Supplementary file 1 — Figure S1: The expression of selected marker genes in different clusters of brain cells. [file CNS-32-e70981-s003.png]
